# Supplementary material for: Surface Grafting of Graphene Flakes with Fluorescent Dyes: A Tailored Functionalization Approach
Source: Nanomaterials (Basel). 2025 Feb 20;15(5):329. doi: 10.3390/nano15050329 (PMC11901827; doi:10.3390/nano15050329)
Supplement: Supplementary file 1 [file nanomaterials-15-00329-s001.zip › nanomaterials-3466312-supplementary.pdf]

nanomaterials

MDPI

Supplementary materials

Surface grafting of graphene flakes with fluorescent dyes: a tailored functionalization approach

Ylea Vlamidis [1,2,*], Carmela Marinelli [2], Aldo Moscardini [3], Paolo Faraci [3], Stefan Heun [1,*] and Stefano Veronesi [1]

[1] NEST, Istituto Nanoscienze–CNR and Scuola Normale Superiore, Piazza San Silvestro 12, 56127 Pisa, Italy; stefano.veronesi@nano.cnr.it
[2] Department of Physical Science, Earth, and Environment, University of Siena, via Roma 56, 53100 Siena, Italy; carmela.marinelli@unisi.it
[3] Scuola Normale Superiore, Laboratorio NEST, Piazza San Silvestro 12, 56127 Pisa, Italy; aldo.moscardini@sns.it (A.M.); paolo.faraci@sns.it (P.F.)
* Correspondence: ylea.vlamidis@unisi.it (Y.V.); stefan.heun@nano.cnr.it (S.H.)

1 .Analytical Techniques and Equipment

1.1. UHPLC-MS Analysis of the Linker Molecule

The linker molecule was analyzed and purified with an Ultra High Performance Liquid Chromatography (UHPLC) system (Shimadzu Nexera). The analyte was identified through a diode-array detector using a wavelength of 227 nm and confirmed with a triple quadrupole mass spectrometer (AB Sciex 3200 QTRAP) equipped with an electrospray ion-ization (ESI) source.

For the analysis, a Kinetex EVO C18 5 µm column (3.0 × 150 mm) was employed. Eluent A was constituted by a 15 mM ammonium acetate buffer (pH 8.0), while Eluent B was a mixture of acetonitrile:eluent A (95:5). The obtained product (~8 mg) was dissolved in a mixture of Eluent A:Eluent B (3:1). The following conditions were employed: flow rate 0.8 mL min-1; gradient separation; first 3 minutes 10% of Eluent B until 90% of B was reached after 15 minutes; this ratio was maintained for a further 4 minutes.

The following mass spectrometer (MS) conditions were employed. Ionization mode: ESI; N2 gas flow 25 mL min-1; ion spray voltage: 5500 V; temperature: 120 °C; ion source gas 1 55.0 mL min-1; ion source gas 2.60 mL min-1; declustering potential: 40 V; entrance potential: 10 V; collision energy: 10 eV.

In Figure S1, the UHPLC-MS analysis of the linker (a) before and (b) after its purification are shown. Using the mix of eluents described before, the retention time of the product was 11.5 ± 0.1 min. The mass spectrum of the product frag-mentation pattern of ions is reported in panel (c).

 https://doi.org/10.3390/xxxxx

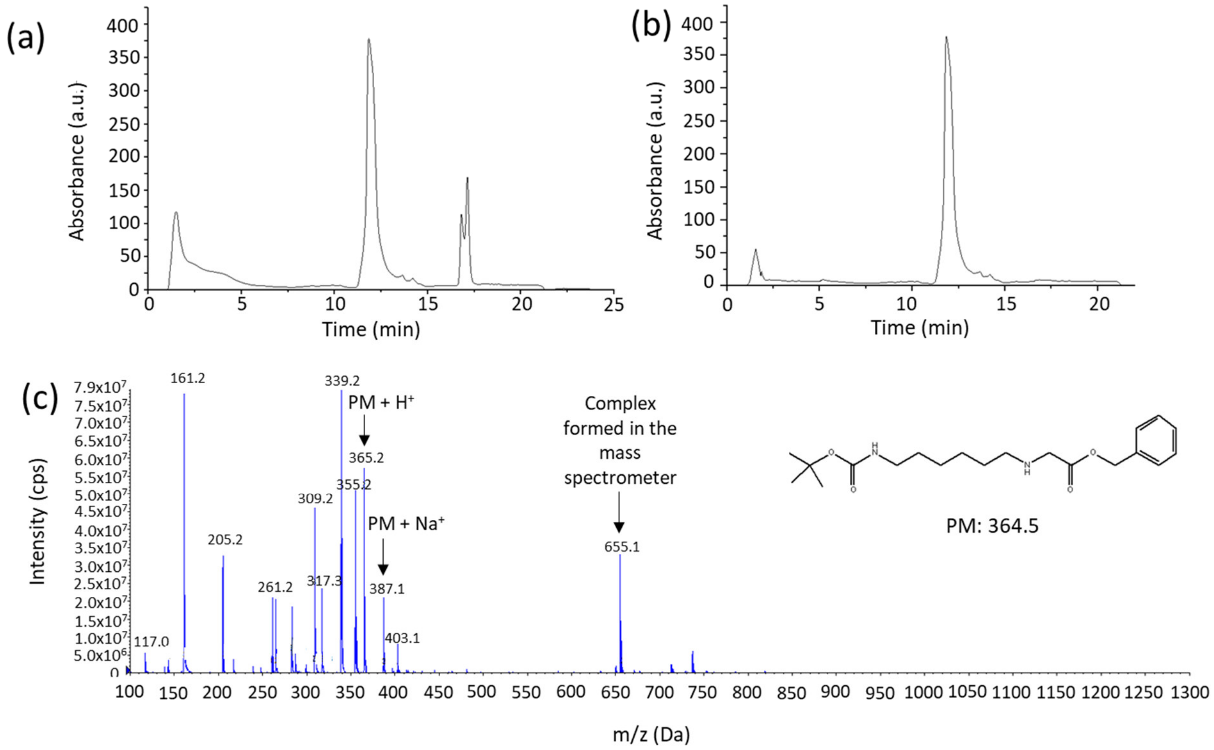

Figure 1. UHPLC-MS analysis of the linker (a) before and (b) after its purification (retention time = 11.5 ± 0.1 min). (c) Mass spectrum of the product fragmentation pattern of ions and molecular structure.

1.2. FT-IR Spectra Analysis

Fourier Transform-Infrared Spectroscopy (FT-IR) was performed with an Agilent Technologies Cary 630 FT-IR Spectrometer to confirm the Boc cleavage in the second step of the functionalization reaction. FT-IR spectra were acquired using Agilent MicroLab FT-IR software. The spectra were recorded between 4000 and 650 cm-1 with a resolution of 4 cm-1.

Figure S2 shows the FT-IR spectra of the cleavage solution before and after amine deprotection. The spectra were acquired after complete evaporation of the dichloromethane to avoid the occurrence of strong absorption bands arising from the solvent.

In the spectra of the cleavage solution, the absorption broad bands around 3500 cm−1 are the characteristic absorption peaks of water molecules or hydroxyl containing molecules (likes alcohols) [1]. The peaks between 1200 and 1300 cm-1 are related to the P=O stretching vibrations of phosphoric acid [2]. The peaks near 1000 cm−1 correspond to the symmetrical stretching of P-O-H, while the peak centered at ~730 cm−1 represent the P-O-H bending mode. [3].

The FT-IR spectrum of the product after Boc cleavage shows peaks between 3600 and 3300 cm-1 which are due to O-H stretching vibrations.1 The new strong bands emerging between 2850 and 2950 cm-1 correspond to the aliphatic C-H stretching vibration4 and suggest that the deprotection reaction is complete. The band at 1730 cm-1 corresponds to the C=O stretch vibration of the amide, while the absorption bands between 1400 and 1300 cm-1 are ascribable to C-H bending mode and O-H in-plane bending vibration [4,5]. The bands due to C-O stretching of tertiary alcohol are observable between 1000 and 1100 cm-

1[6]. Eventually, the band at ~710 cm-1 is caused by the O-H out-of-plane bending vibration [7].

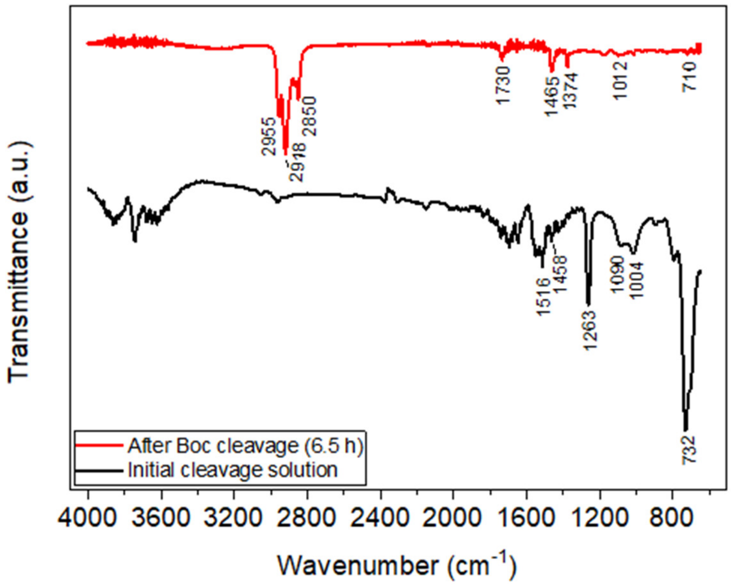

Figure S2. FT-IR spectra (linear scale) acquired for the cleavage solution (phosphoric acid /CH2Cl2/H2O, black line) and at the end of the reaction (t = 6.5 h, red line), demonstrating the successful deprotection reaction. The spectra were acquired after complete evaporation of CH2Cl2.

2. Further fluorescence characterization of the graphene flakes labeled with fluorophores

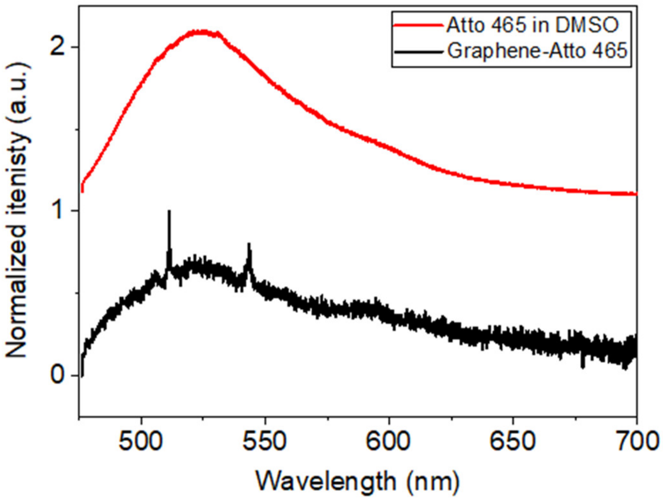

Figure S3. PL spectra acquired with at 473 nm excitation: comparison between the normalized spectra of Atto 465 NSH in DMSO (1 µg µL-1) drop-casted on a glass slice (red line) and graphene flakes functionalized with the same fluorophore (black line).

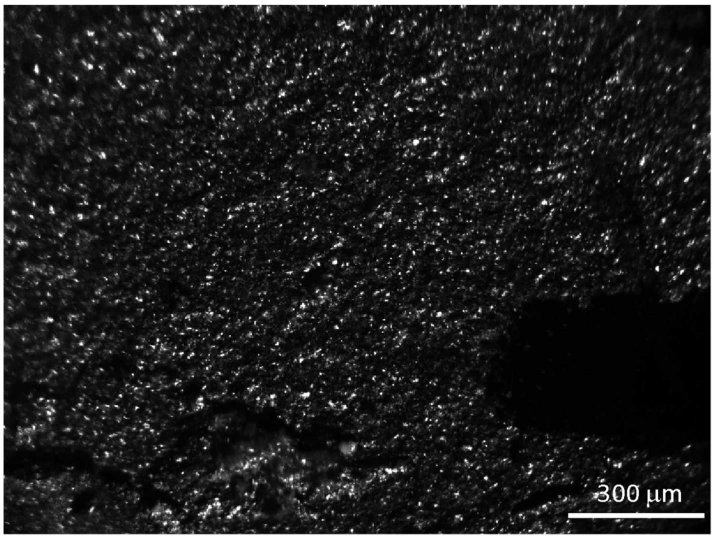

Figure S4. Optical image of the graphene flakes functionalized with Atto 465 under blue LED light illumination acquired with a 5x objective. The sample is fully covered by functionalized flakes, except for the lower right corner of the image where a scratch has removed the flakes and no fluorescence is detected.

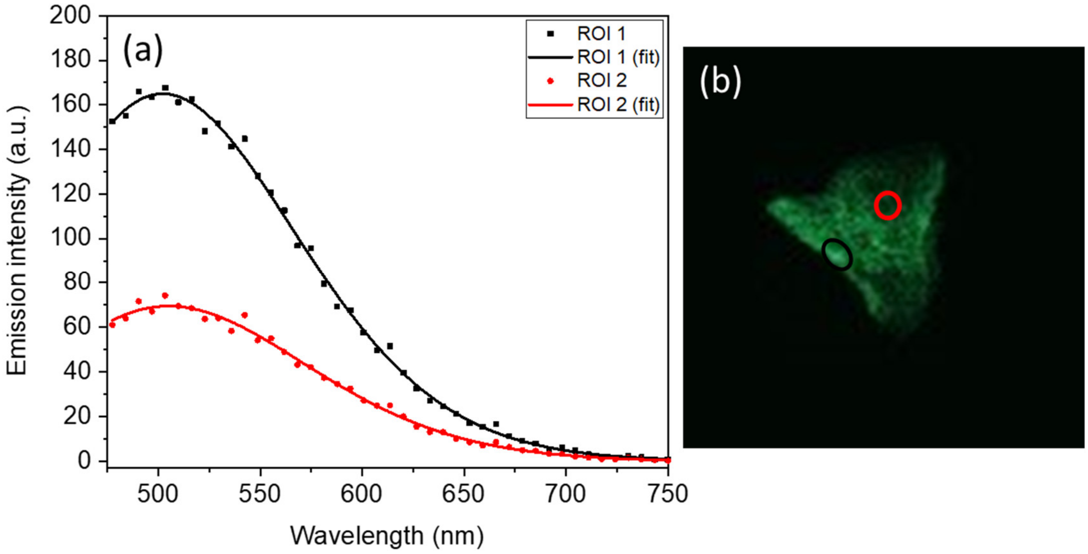

Figure S5. (a) Fluorescence spectra of Atto 465-labeled graphene acquired with a confocal microscope in two regions of interest (ROI) shown in panel (b). Excitation wavelength: 470 nm. The luminescence intensity, indicating the degree of functionalization, is not homogeneous across the flakes. This variation is attributed to the typical defect distribution, which is higher at the edges of the graphene flakes. However, it is noteworthy that functionalization also extends to the basal planes, contributing to the overall luminescence pattern.

2.1. Lifetime Measurements

For the lifetime measurements of the graphene flakes functionalized with dyes, the total fluorescence emitted by the samples was collected, whereas the control samples consisted of the pure fluorophores dissolved in chloroform (3 µg µL-1). Calibration of the

system was performed using the lifetime decay of a stock solution of fluorescein in ethanol (100 µM) diluted 1:500 in 0.1 M NaOH (pH =11). Excitation was performed at a wavelength of 488 nm, and fluorescence was collected in the range 400–570 nm.

Similarly to what already observed in the case of graphene flakes labeled with Atto 465, both graphene samples functionalized with Atto 425 and Atto 633 exhibited double exponential fluorescence decay curves, whereas typical first order reactions were observed for the free fluorophores in solution (refer to Figure S6). The average lifetimes obtained for each sample analyzed are reported in Table S1.

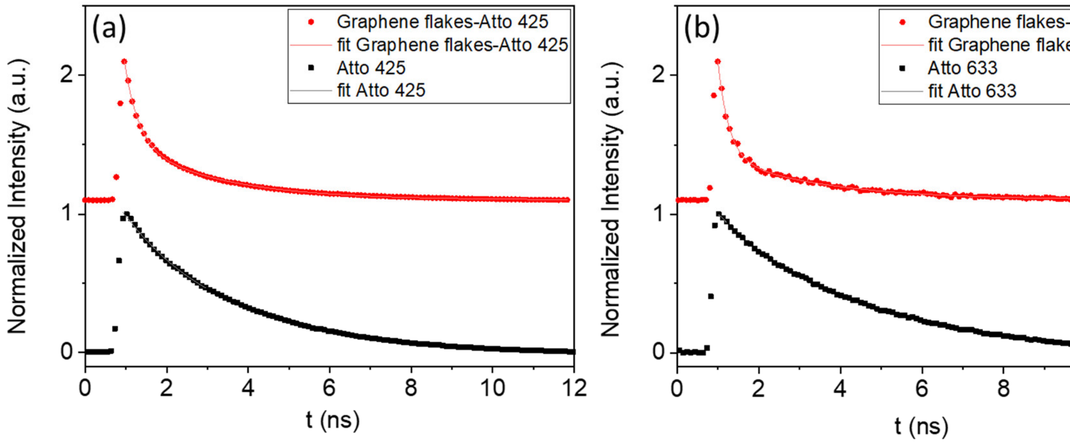

Figure S6. Normalized fluorescence decay with fitting curves of the samples functionalized with (a) Atto 425 and (b) Atto 633 compared to those typical of the pure fluorophores in chloroform (3 µg µL-1), obtained from FLIM images.

Table S1. Lifetimes obtained for graphene flakes functionalized with Atto 465, Atto 425, and Atto 633 and for the respective pure fluorophores in chloroform (3 µg µL-1). Average value ± standard deviation from five measurements

Sample	τ (ns)	τ_1 (ns)	τ_2 (ns)
Atto 465 (pure)	3.5±0.1		
Atto 465-graphene flakes		0.8±0.1	2.9±0.1
Atto 425 (pure)	2.8±0.1		
Atto 425-graphene flakes		0.5±0.1	2.7±0.1
Atto 633 (pure)	3.7±0.1		
Atto 633-graphene flakes		0.5±0.1	2.6±0.1

References

Ahmed Y.M.Z., El-Sheikh S.M., Zaki Z.I. Changes in hydroxyapatite powder properties via heat treatment. Bull. Mater. Sci. 2015, 38, 1807–1819. https://doi.org/10.1007/s12034-015-1047-0.

2 Rudolph W.W. Raman- and infrared-spectroscopic investigations of dilute aqueous phosphoric acid solutions, Dalt. Trans. 2010, 39, 9642–9653. https://doi.org/10.1039/C0DT00417K

3 Abifarin J.K., Obada D.O., Dauda E.T., Dodoo-Arhin D. Experimental data on the characterization of hydroxyapatite synthesized from biowastes. Data Brief 2019, 26 104485. https://doi.org/10.1016/j.dib.2019.104485.

4 Mecozzi M., Pietroletti M., Scarpiniti M., Acquistucci R., Conti M.E. Monitoring of marine mucilage formation in italian seas investigated by infrared spectroscopy and independent component analysis. Environ. Monit. Assess. 2012, 184, 6025–6036. https://doi.org/10.1007/s10661-011-2400-4.

5 Shao D., Wei Q., Microwave-assisted rapid preparation of nano-ZnO/Ag composite functionalized polyester nonwoven membrane for improving its UV shielding and antibacterial properties. Materials 2018, 11. https://doi.org/10.3390/ma11081412.

6 Tretinnikov O.N., Zagorskaya S.A., Determination of the degree of crystallinity of poly(vinyl alcohol) by FTIR spectroscopy, J. Appl. Spectrosc. 2012, 79, 521–526. https://doi.org/10.1007/s10812-012-9634-y.

7 Bishop J., Murad E., Dyar M.D. The influence of octahedral and tetrahedral cation substitution on the structure of smectites and serpentines as observed through infrared spectroscopy. Clay Minerals 2002, 37, 617–628. https://doi.org/10.1180/0009855023740064.
